# Integrated transcriptome and network analysis identifies EZH2/CCNB1/PPARG as prognostic factors in breast cancer

**DOI:** 10.3389/fgene.2022.1117081

**Published:** 2023-01-11

**Authors:** Yalun Li, Gang Chen, Kun Zhang, Jianqiao Cao, Huishan Zhao, Yizi Cong, Guangdong Qiao

**Affiliations:** ^1^ Department of Breast Surgery, The Affiliated Yantai Yuhuangding Hospital of Qingdao University, Yantai, Shandong, China; ^2^ Reproductive Medicine Centre, The Affiliated Yantai Yuhuangding Hospital of Qingdao University, Yantai, Shandong, China

**Keywords:** breast cancer, bioinformatics, prognosis biomarker, GEO, TCGA

## Abstract

Breast cancer (BC) has high morbidity, with significant relapse and mortality rates in women worldwide. Therefore, further exploration of its pathogenesis is of great significance. This study selected therapy genes and possible biomarkers to predict BC using bioinformatic methods. To this end, the study examined 21 healthy breasts along with 457 BC tissues in two Gene Expression Omnibus (GEO) datasets and then identified differentially expressed genes (DEGs). Survival-associated DEGs were screened using the Kaplan–Meier curve. Based on Gene Ontology (GO) annotation, survival-associated DEGs were mostly associated with cell division and cellular response to hormone stimulus. The enriched Kyoto Encyclopedia of Gene and Genome (KEGG) pathway was mostly correlated with cell cycle and tyrosine metabolism. Using overlapped survival-associated DEGs, a survival-associated PPI network was constructed. PPI analysis revealed three hub genes (*EZH2*, *CCNB1*, and *PPARG*) by their degree of connection. These hub genes were confirmed using The Cancer Genome Atlas (TCGA)-BRCA dataset and BC tissue samples. Through Gene Set Enrichment Analysis (GSEA), the molecular mechanism of the potential therapy and prognostic genes were evaluated. Thus, hub genes were shown to be associated with KEGG_CELL_CYCLE and VANTVEER_BREAST_CANCER_POOR_PROGNOSIS gene sets. Finally, based on integrated bioinformatics analysis, this study identified three hub genes as possible prognostic biomarkers and therapeutic targets for BC. The results obtained further understanding of the underground molecular mechanisms related to BC occurrence and prognostic outcomes.

## Introduction

Breast cancer (BC) is highly prevalent and despite therapeutic advances, has the highest cancer-related mortality in women (Siegel et al., 2020). Although long-term all-round radiation therapy can enhance patient survival, there are undiscovered regional lymphatics within the radiation zone, and still, over 30% of BC patients develop distant metastasis, causing a high fatality rate (Moreno et al., 2017; Zhao et al., 2019). Therefore, exploring the molecular mechanisms associated with BC prognosis and treatment is of great significance. The Cancer Genome Atlas (TCGA, https://portal.gdc.cancer.gov/) and Gene Expression Omnibus (GEO, https://www.ncbi.nlm.nih.gov/geo) databases have high flux and efficiency and have been widely applied in various disease research gene chip platforms, including for lung (Cai et al., 2021), kidney (Xu et al., 2021), and colon cancers. In recent years, some bioinformatics assays on BC have been reported and different chip databases have been selected to investigate the potential mechanisms.

The Nottingham Prognosis Index (NIP) has been adopted for investigating gene features (Zhou et al., 2022), and machine learning modules have been utilized to select and confirm the prediction accuracy of biomarkers (Tabl et al., 2019). In this preliminary study, the integrated bioinformatics method is applied. We downloaded two gene chip datasets (GSE31448 and GSE42568) from the GEO database, consisting of 457 BC and 21 non-carcinoma mammary tissue samples. Then, differentially expressed genes (DEGs) were identified by adopting R statistical software from the aforementioned data, while the intersection of the aforementioned maps was obtained by Venn diagram. The survival of the DEGs was investigated using online KM-plotter. Online analysis using The Database for Annotation, Visualization and Integrated Discovery (DAVID), KOBAS, Gene Ontology (GO) analysis, and the Kyoto Encyclopedia of Gene and Genome (KEGG) pathway analysis was conducted to establish survival-associated DEGs. A survival-associated protein–protein interaction (PPI) network was constructed on the basis of String online tools and visualized by Cytoscape software and the cytoHubba plugin to detect hub genes. The top three degrees of hub genes (*EZH2*, *CCNB1*, and *PPARG*) were then selected and the TCGA_BRCA dataset was utilized to confirm them. Gene Set Enrichment Analysis (GSEA) was conducted to study the potential mechanisms of these hub genes. Having obtained credible findings, BC samples were used for verification of hub genes, with consistent results obtained. This work identified three BC survival-associated biomarkers, which help us to understand the molecular mechanisms of BC development.

## Materials and methods

### Microarray data

The GEO database (https://www.ncbi.nlm.nih.gov/geo/) is a public and freely available microarray database, which can be adopted in platform records and gene expression datasets. GSE31448 and GSE42568, the gene expression files of series matrix, were downloaded from the database. GSE31448 is based on the GPL570 platform ([HG-U133_Plus_2] Affymetrix Human Genome U133 Plus 2.0 Array) and includes 353 BC tissues and 4 normal breast tissues. GSE42568 is based on the same platform as GSE31148, including 104 BC and 17 normal breast tissues. The downloaded dataset was used with log2 transformation and Z-score standardization.

### Screening for DEGs

R (version 4.1.1) limma (version 3.30.0) was employed to identify DEGs in BC compared with non-carcinoma samples with thresholds of |logFC| ≥ 1.0 and false discovery rate (FDR) < 0.05. Online Venn software (http://www.biovenn.nl/index.php) was then used to acquire intersected DEGs in two datasets.

### Survival analysis of DEGs

To identify survival-associated DEGs, we employed the Kaplan–Meier plotter (http://kmplot.com/analysis/) for analysis. DEGs were cut into two departs based on the median expression levels, and the logrank *p* ≤ 0.05 seemed to be of statistical significance.

### GO and KEGG enrichment analysis of survival-associated DEGs

As a general analysis, GO enrichments consisted of biological processes (BP), cellular components (CC), and molecular functions (MF). In addition, KEGG pathway enrichment analysis was employed to investigate key pathways for the DEGs. The GO enrichment was conducted with DAVID (2021 Update, https://david.ncifcrf.gov/), while the KEGG pathway enrichment for DEGs was conducted with the online tool KOBAS 3.0 (http://kobas.cbi.pku.edu.cn/). FDR<0.05 and the counts numbering over 2 were selected as a statistically significant enrichment.

### Constructing the survival-associated PPI network and identifying the hub gene

The survival-associated PPI network was constructed with String online tools. Cytoscape (version 3.9.1) software was then adopted to visualize this network. Hub genes were predicted with the use of the cytoHubba plugin. A network combination score of more than 0.9 was set as the cutoff.

### Validating the hub genes in the TCGA database

To obtain the TCGA_BRCA dataset from the TCGA database, we employed the R software TCGA-Assembler (version 2.0.6) package. DEGs were investigated with the R software DESeq package. The three hub gene functions were further enriched with GSEA (version 4.1.0).

### Tissue samples of BC patients

Seven BC cases admitted to the Department of Breast Surgery, Affiliated Yantai Yuhuangding Hospital of Qingdao University, were enrolled for the current work. Each was required to provide informed consent before participation. Our study was approved by the Institutional Review Board of Yantai Yuhuangding Hospital (Certificate number: 2022-404).

### RNA isolation and Q-PCR

Using the TRIzol reagent, total tissue RNAs were extracted following the specific protocols (Shandong Sparkjade Biotechnology Co., Ltd.) of the previous study (Chen et al., 2021). Finally, the 2^−ΔΔCT^ method was employed to determine mRNA expression (Gomez-Rodriguez et al., 2008). Primer sequences are shown in Table1.

**TABLE 1:** Primer sequences of three hub genes used for Q-PCR.

Gene	Forward primer (5′-3′)	Reverse primer (5′-3′)
*EZH2*	AGC​AGT​GCC​CGT​GCT​ACC​T	ATG​GTG​CCA​GCA​ATA​GAT​GCT​T
*CCNB1*	TGA​AGA​AAT​GTA​CCC​TCC​AGA​AAT​T	CTC​CGA​AGG​AAG​TGC​AAA​GG
*PPARG*	TCA​TGC​TTG​TGA​AGG​ATG​CAA	ATC​CCC​ACT​GCA​AGG​CAT​T
*GAPDH*	CAT​GTT​CGT​CAT​GGG​TGT​GAA	GGC​ATG​GAC​TGT​GGT​CAT​GAG

### Statistical analysis

R statistics software was employed for statistical analysis. In addition, data were suggested as the mean ± standard deviation (SD) of the dataset. By applying GraphPad Prism 5 software (GraphPad Inc., San Diego, CA, United States), the hub genes from the TCGA dataset and Q-PCR statistical analysis were analyzed with paired Student’s t-test (two-tailed). *p* ≤ 0.05 was thought to be significantly different.

## Results

### Identification of DEGs from GEO profiles

**FIGURE 1:**
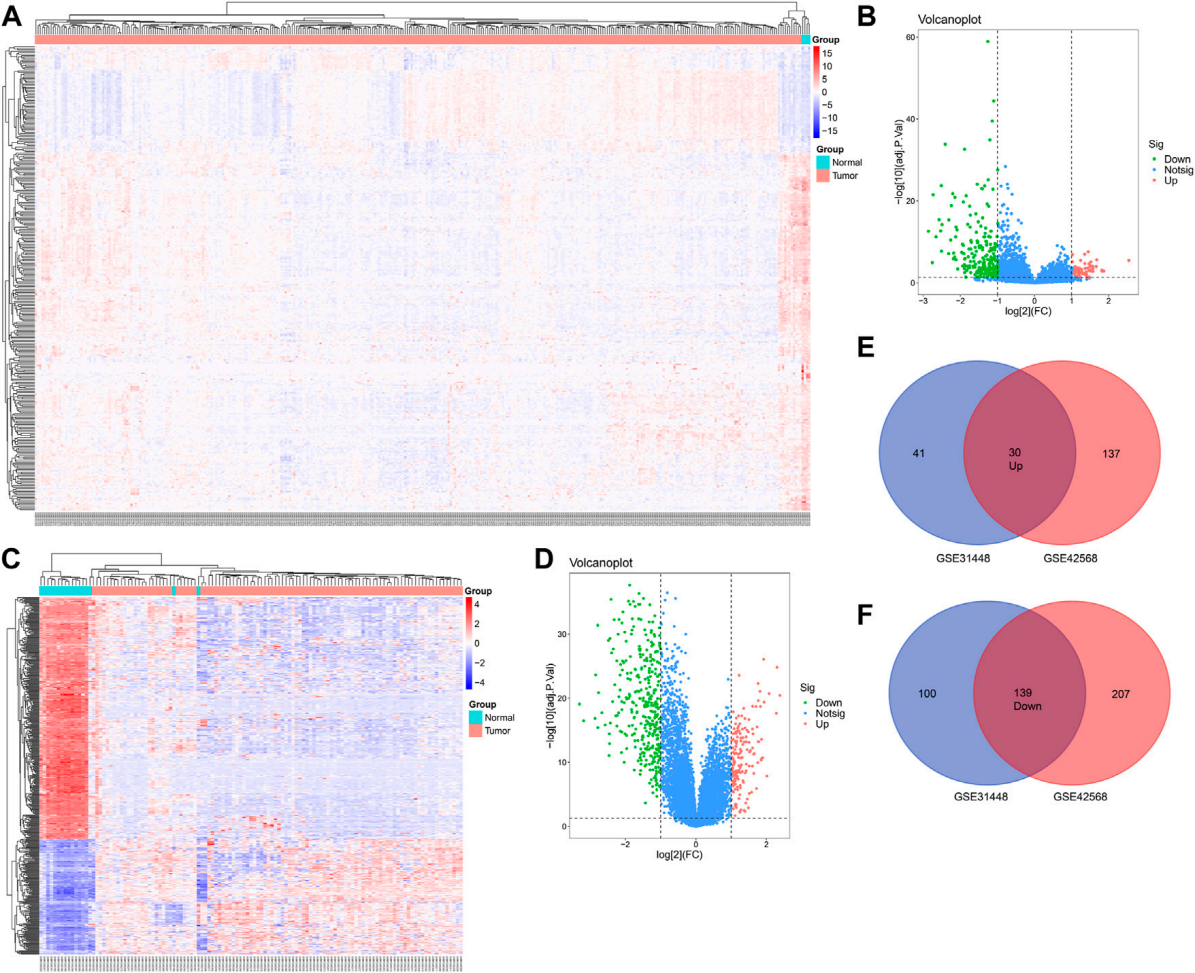
Differentially expressed genes in BC patients compared with the normal group. **(A,B)** Heatmap of differentially expressed mRNAs in BC, based on GSE31448 and GSE42568. **(C,D)** Volcano map of all mRNAs in GSE31448 and GSE42568. **(E,F)** Overlapped DEGs between GSE31448 and GSE42568.

A total of 71 upregulated and 239 downregulated genes were selected from GSE31448 and 167 upregulated and 349 downregulated genes from GSE42568 (|logFC|≥ 1.0 and FDR< 0.05). The heat map and volcano plot of the DEGs are displayed in Figures 1A–D. The overlapped DEGs (30 with upregulation and 139 with downregulation) were chosen by the online Venn tool (Figures 1E, F).

### Survival analysis of DEGs

To identify the survival-associated DEGs, we adopted the Kaplan–Meier plotter (http://kmplot.com/analysis/), and found 24 upregulated and 79 downregulated DEGs that were consistent with our expectations (Supplementary Figures S1, S2).

### GO and KEGG enrichment analyses of survival-associated DEGs

**FIGURE 2:**
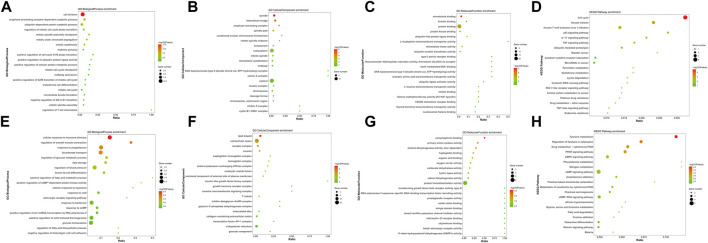
GO and KEGG enrichment analyses of survival-associated DEGs. **(A–C)** GO analysis of upregulated survival-associated DEGs. **(E–G)** GO analysis of down-regulated survival-associated DEGs. **(D,H)** KEGG analysis of up- and downregulated survival-associated DEGs.

To investigate the biological functions and pathways of the 103 integrated DEGs (24 upregulated and 79 downregulated) in BC, GO and KEGG enrichment analyses were conducted using the DAVID and KOBAS online analysis tools. The upregulated DEGs were mostly engaged in the “cell division” and “spindle,” “microtubule binding” in BP and CC, as well as the MF aspect (Figures 2A–C). The downregulated DEGs were mostly enriched in “cellular response to hormone stimulus,” “lipid droplet,” and “norepinephrine binding” in BP and CC, as well as the MF segment (Figures 2E–G). The KEGG pathway “Cell cycle” and “Tyrosine metabolism” were mostly enriched for the up- and down-regulated DEGs (Figures 2D, H).

### Constructing survival-associated PPI network and identifying hub genes

**FIGURE 3:**
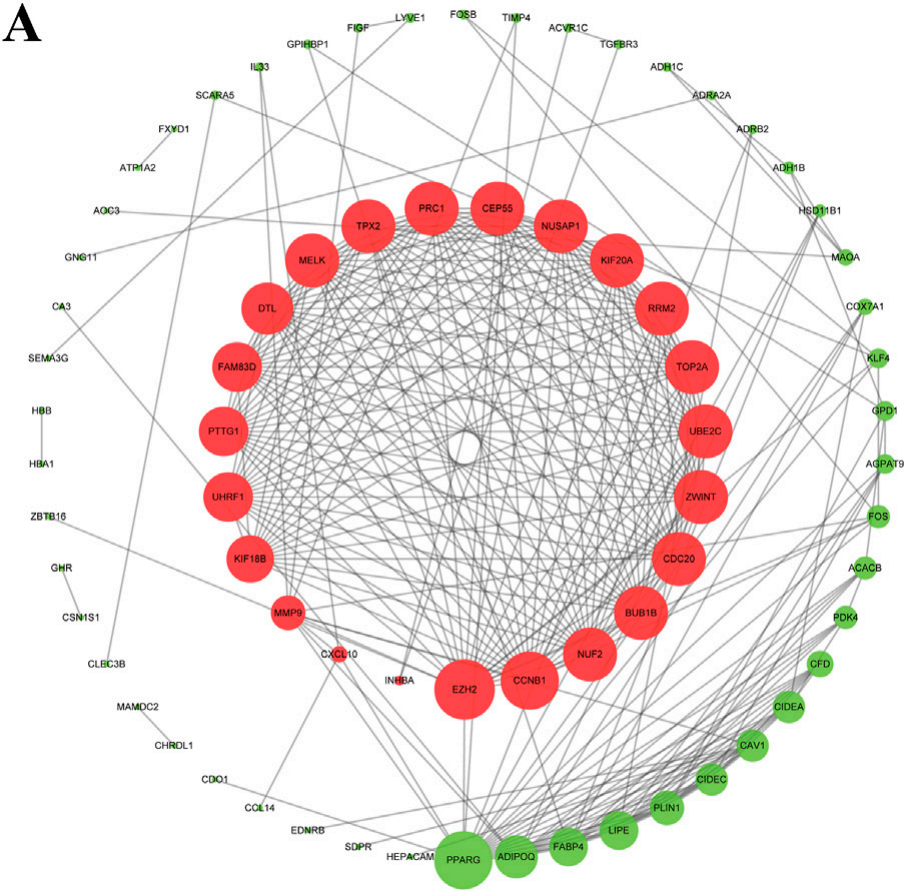
Survival-associated network in BC. The red and green nodes represent up- and downregulated DEGs, respectively.

The String online tool was used to construct the survival-related PPI network. The graph was then visualized using Cytoscape software. The top three DEGs with the highest degree were selected by the Cytohubba plugin, including two with upregulation (*EZH2* and *CCNB1*) and one with downregulation (*PPARG*) (Figure 3).

### Validating the hub genes using the TCGA database and BC samples

**FIGURE 4:**
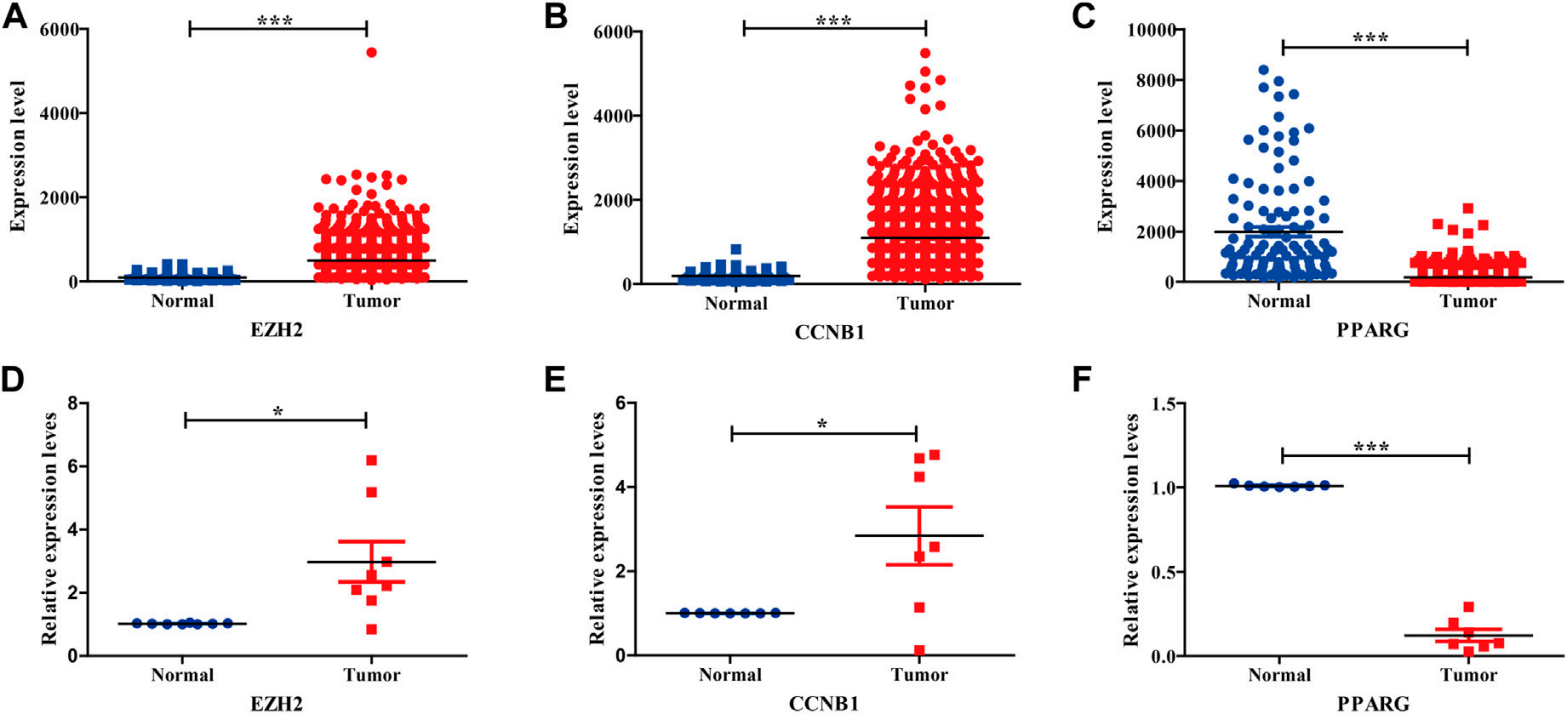
Expression validation of three hub genes in BC compared with adjacent tissues from TCGA datasets and BC tissues. **(A–C)** Relative expression levels of three hub genes in TCGA database. **(D–F)** Relative expression levels of three hub genes in BC tissues.

To further survey the three hub genes, the TCGA_BRCA dataset was acquired from the TCGA database. Our results indicated that three hub genes showed obvious differences between tumor and normal tissues, conforming to the aforementioned GEO results (Figures 4A–C). The three hub genes were also validated by tissue samples from BC patients, consistent with the aforementioned GEO and TCGA database analyses (Figures 4D–F). Table 1 displays Q-PCR primer sequences for hub genes.

### GSEA of hub genes

**FIGURE 5:**
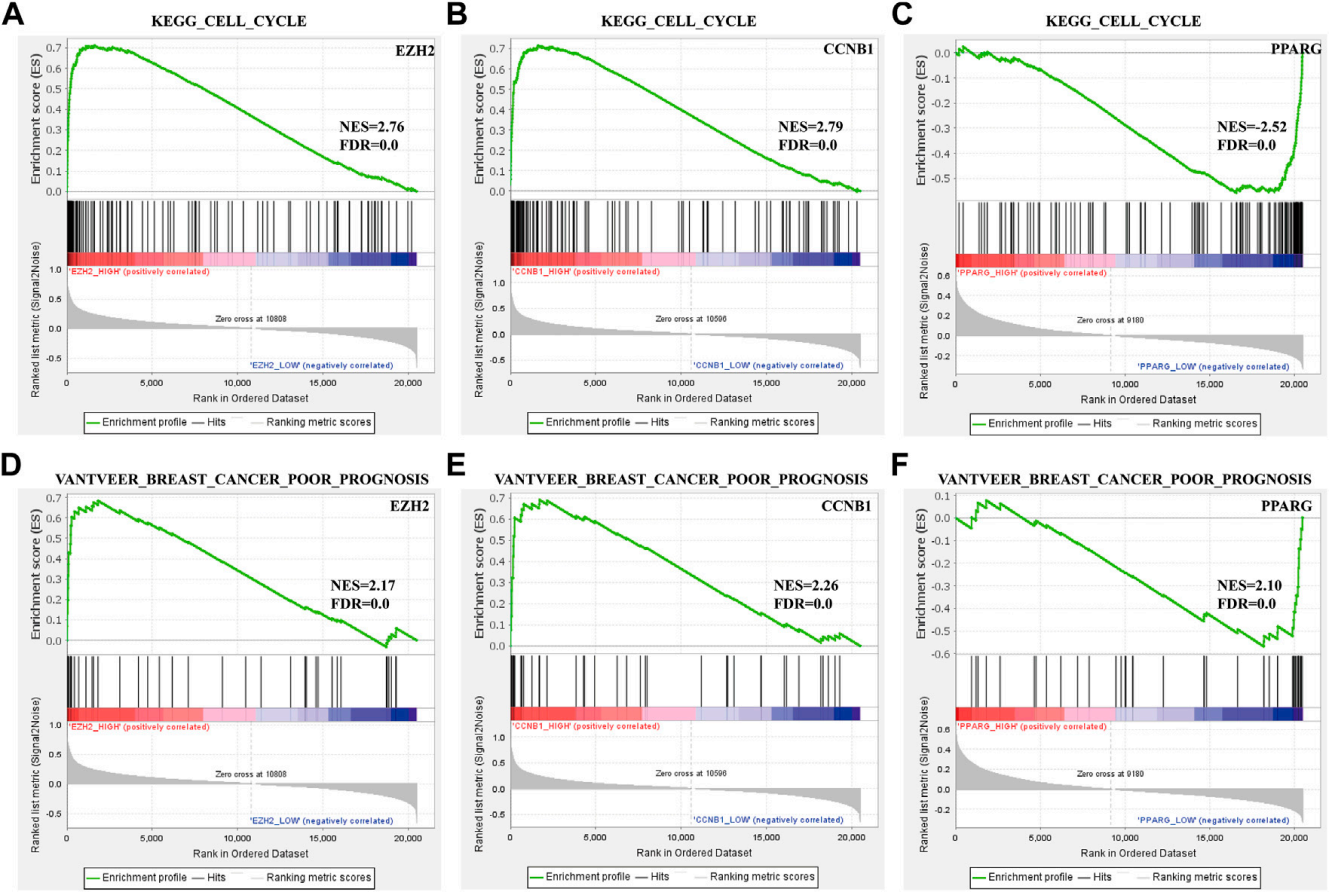
Enrichment plots from GSEA. **(A–C)** ‘VANTVEER_BREAST_CANCER_POOR_PROGNOSIS’ and **(D–F)** ‘KEGG_CELL_CYCLE’ gene sets enrichment plots of three hub genes.

To identify the prognosis and mechanism underlying the BC hub genes, GSEA was performed. The TCGA_BRCA dataset was divided into two segments from the middle levels. We analyzed the ‘VANTVEER_BREAST_CANCER_POOR_PROGNOSIS’ and ‘KEGG_CELL_CYCLE’ gene sets. The findings suggested that CCNB1 was positively related to a poor prognosis of BC (Figure 5).

## Discussion

The incidence of BC among female cancer patients is approximately 30%,—the highest for cancer—and its mortality rate ranks second. Similarly, the incidence of BC in China is up to 17.07%, which also ranks first but with a younger trend. BC has the highest fatality of all cancers in female patients worldwide, representing a major mortality factor for women (Cheng et al., 2020). Based on the newest cancer statistics, there will be 51,400 ductal carcinoma *in situ* cases diagnosed in BC women in the USA in 2022 (Siegel et al., 2022). Despite advances in diagnosis, BC is a huge threat to women due to the rapid progress of drug resistance and a lack of therapeutic target methods. Consequently, a sensitive and special biomarker for BC is urgently needed.

Our study involved two gene chips based on the GEO database. A total of 169 DEGs (30 up- and 139 downregulated) were selected. Due to the high BE mortality rate, early diagnostic markers play a vital role in favorable prognoses. Although many genes are recognized as specific diagnostic indicators for BC, clinical evidence is lacking. In this study, 105 (24 with upregulation and 81 with downregulation) survival-associated DEGs were identified by GEO database analysis. Based on GO and KEGG enrichment, upregulated survival-associated DEGs were the most enriched in “cell division” and “spindle,” “microtubule binding,” and “cell cycle” in, respectively, BP, CC, and MF segments and KEGG pathways. Downregulated survival-associated DEGs were most enriched in “cellular response to hormone stimulus” and “lipid droplet,” “norepinephrine binding,” and “Tyrosine metabolism” in, respectively, BP, CC, and MF terms and KEGG pathways. To determine which gene is the pivotal regulator for BC, a survival-associated PPI network was constructed, with three hub genes (*EZH2*, *CCNB1*, and *PPARG*) chosen using the cytoHubba plugin. These three hub genes were verified by the TCGA_BRCA dataset and tissue samples from BC patients. To investigate the underlying mechanism of hub genes, GSEA was conducted. VANTVEER_BREAST_CANCER_POOR_PROGNOSIS’ and ‘CELL_CYCLE’ gene sets were positively correlated with the three hub genes. We recognized these three genes to be the hub genes related to poor prognoses of BC. Multivariate and univariate analyses of genetic association studies and genome-wide association studies have received a remarkable attention as they improve analytical precision (Puddu et al., 1997; Dimou et al., 2018) From the multivariate and univariate analyses, the aforementioned genes are associated with patients’ clinical features, such as the TNM-based stage (Supplementary Figures S3, S4). The details of these genes are as follows.

The enhancer of zeste homolog 2 (EZH2) is a member of the polycomb gene (PcG) family. EZH2 is an important factor in regulating cell autophagy and apoptosis (Yao et al., 2016), which can promote DNA damage repair and the cell cycle (Nutt et al., 2020). This gene not only has a diversified role in cell function but is also related to many diseases, including cancer. An aberrant expression of EZH2 enhances cell proliferation, which may cause tumor development. Others have reported that EZH2 was correlated with poor clinical outcomes for prostate cancer (Varambally et al., 2002). More evidence shows that EZH2 is strongly denoted in numerous tumors, including esophageal cancer (Qiu et al., 2020), gastric cancer (Gan et al., 2018), and endometrial carcinoma (Krill et al., 2020), which also contains BC (Bachmann et al., 2006). An *in vivo* mice model experiment demonstrated that EZH2 is positively related to the lymph nodes and distant metastases (Zingg et al., 2015). EZH2 can thus act as a prognosis biomarker for BC. Cyclin B1 (CCNB1) is a cell cycle regulator which can form complexes with CCNB2 and CDK1 to regulate the G2/M phases of the cell cycle. The dysregulation of CCNB1 is associated with aberrant cell growth. Moreover, the high expression of CCNB1 shows positive association with unfavorable outcomes for non-small-cell lung, breast, gastric, and esophageal cancers (Roskoski 2019). Nevertheless, its precise functions in BC need elucidation. As the nuclear receptor, peroxisome proliferator-activated receptor gamma (PPARG) can antagonize the transcription of immune and inflammatory factors and is also involved in the energy metabolism (Medina-Gomez et al., 2007), adipocyte differentiation, and inflammation (Mirza et al., 2019; Villa et al., 2020). Studies have revealed that PPARG exerts a vital role in a number of tumors, including colorectal (Villa, Parra, Feitosa, Camargo, Machado, Tirapelli, Rocha and Feres 2020), renal (Fujita et al., 2011), and bladder cancers (Plissonnier et al., 2010). Some argue that PPARG expression inhibits the Wnt/beta-catenin pathway (Vallee et al., 2019) and is positively related to patient prognosis (Ogino et al., 2009).

To sum up, our study has identified three hub genes (*EZH2*, *CCNB1*, and *PPARG*) related to the prognosis and therapeutic target of BC based on comprehensive gene chip datasets combined with bioinformatic analyses. These findings provide us with a high perspicacity for gene therapy of BC. However, due to the limitations of the present study, more cell research and experiments are essential to study the roles of hub genes in BC and more experiments need to be conducted for validation.

## Data Availability

The original contributions presented in the study are included in the article/Supplementary Material; further inquiries can be directed to the corresponding authors.

## Supplementary material

Supplementary Figure S1 Survival-associated upregulated DEGs.

Supplementary Figure S2 Survival-associated downregulated DEGs.

Supplementary Figure S3 Univariate analysis forest of hub genes.

Supplementary Figure S4 Multivariate analysis forest of hub genes.
