# Supplementary figures and images for: Integrated transcriptome and network analysis identifies EZH2/CCNB1/PPARG as prognostic factors in breast cancer
Source: Front Genet. 2023 Jan 11;13:1117081. doi: 10.3389/fgene.2022.1117081 (PMC9873965; doi:10.3389/fgene.2022.1117081)

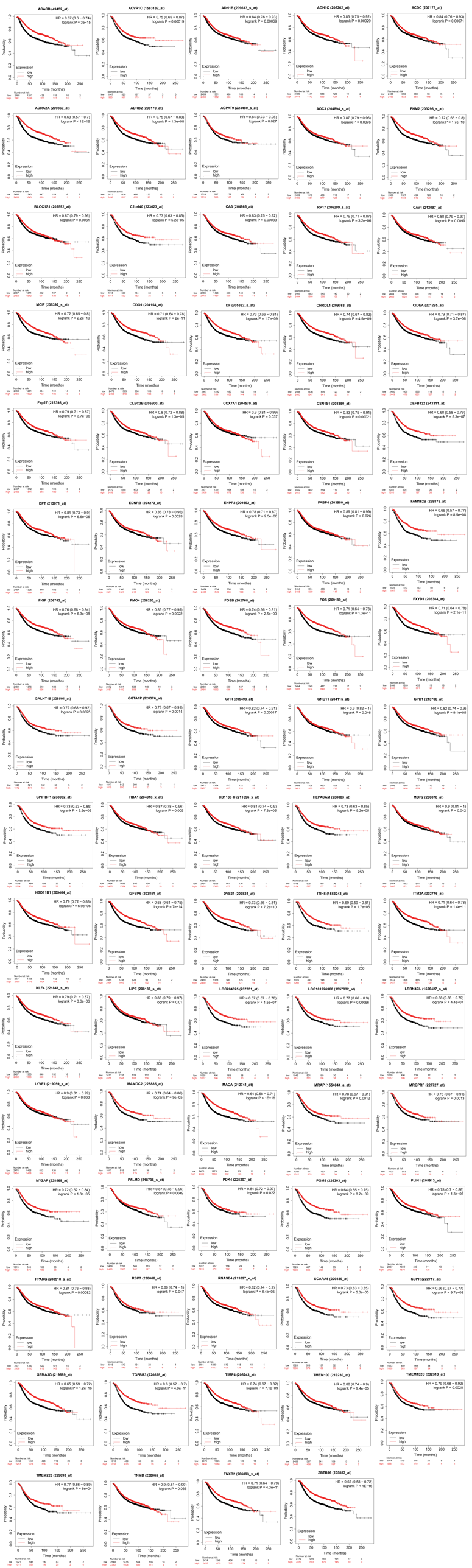

Supplement: Supplementary file 1 [file DataSheet2.PDF]

|           | <b>pvalue</b> | <b>Hazard ratio</b> |
|-----------|---------------|---------------------|
| age       | <0.001        | 1.034(1.019–1.049)  |
| gender    | 0.458         | 0.473(0.065–3.418)  |
| stage     | 0.051         | 1.652(0.998–2.734)  |
| T         | 0.826         | 1.033(0.771–1.384)  |
| M         | 0.268         | 1.592(0.699–3.628)  |
| N         | 0.204         | 1.205(0.904–1.607)  |
| riskScore | <0.001        | 1.812(1.342–2.445)  |

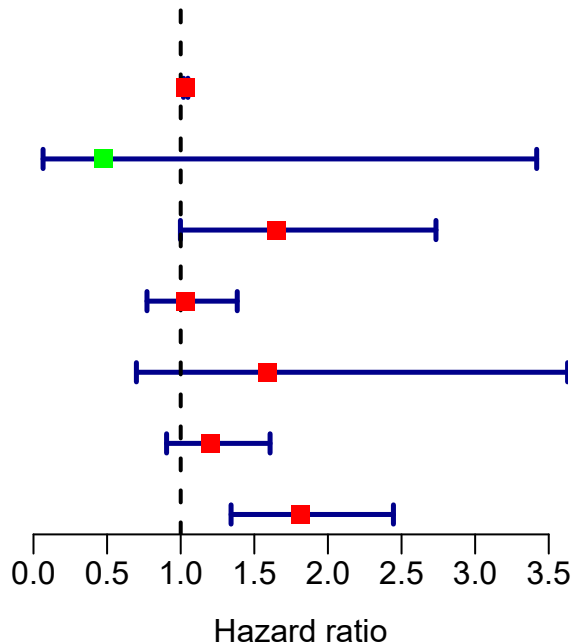

Supplement: Supplementary file 2 [file DataSheet4.PDF]

|           | <b>pvalue</b> | <b>Hazard ratio</b> |
|-----------|---------------|---------------------|
| age       | <0.001        | 1.035(1.020–1.050)  |
| gender    | 0.867         | 0.845(0.118–6.059)  |
| stage     | <0.001        | 2.176(1.721–2.750)  |
| T         | <0.001        | 1.546(1.247–1.917)  |
| M         | <0.001        | 6.401(3.589–11.419) |
| N         | <0.001        | 1.743(1.443–2.106)  |
| riskScore | <0.001        | 1.847(1.393–2.449)  |

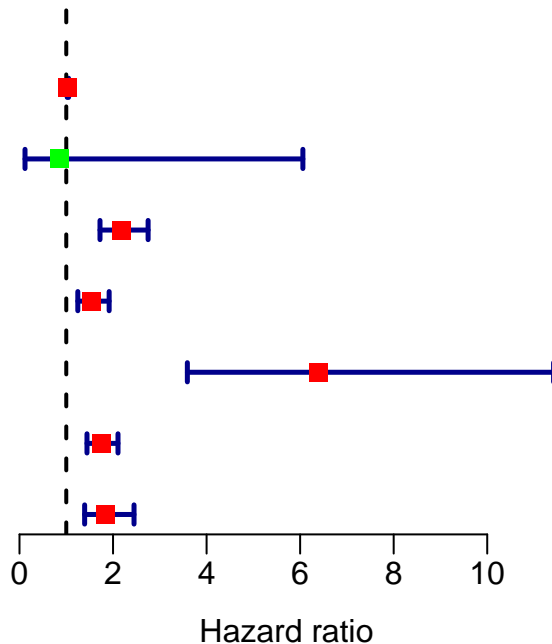

Supplement: Supplementary file 3 [file DataSheet3.PDF]

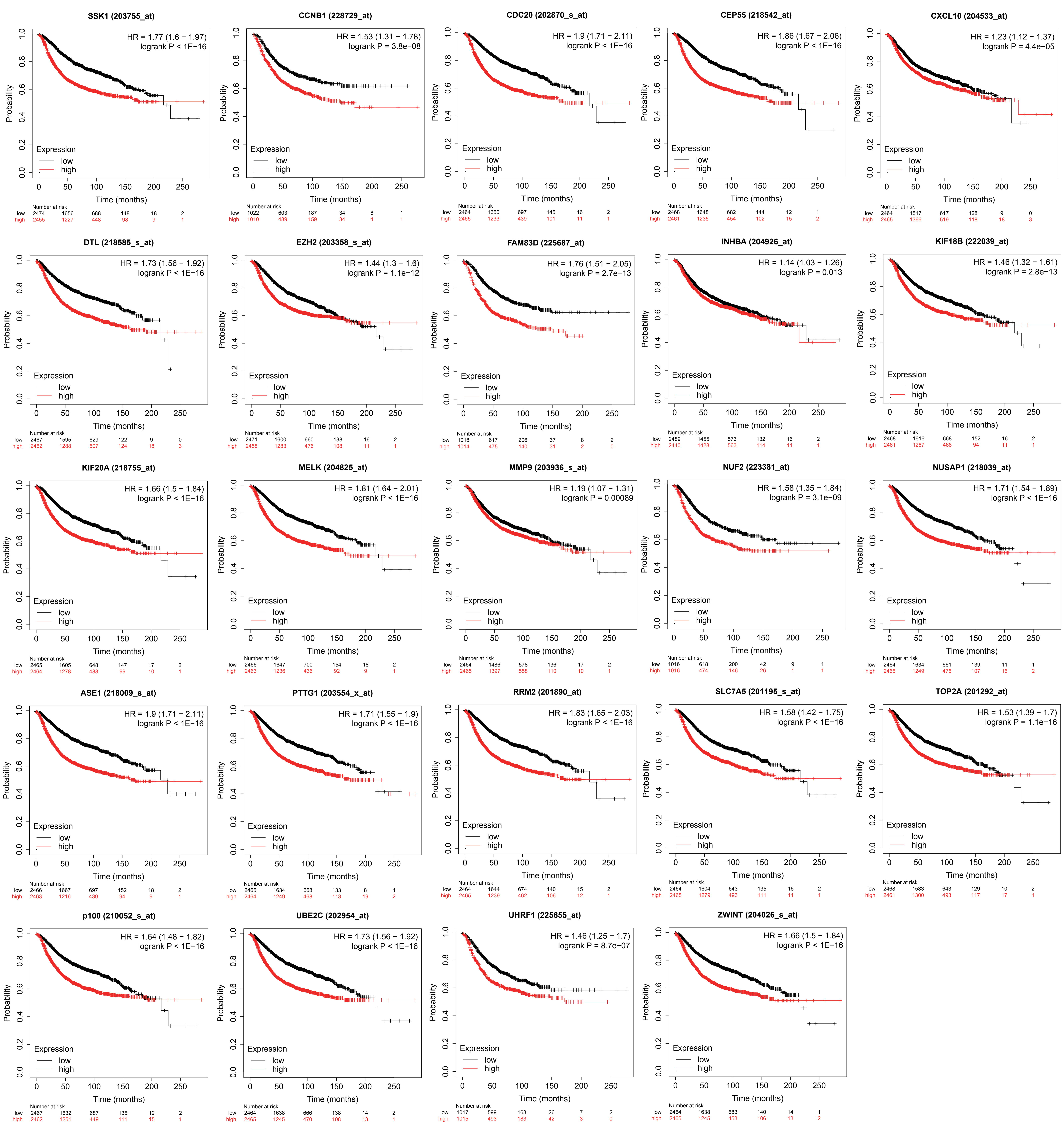

Supplement: Supplementary file 4 [file DataSheet1.PDF]

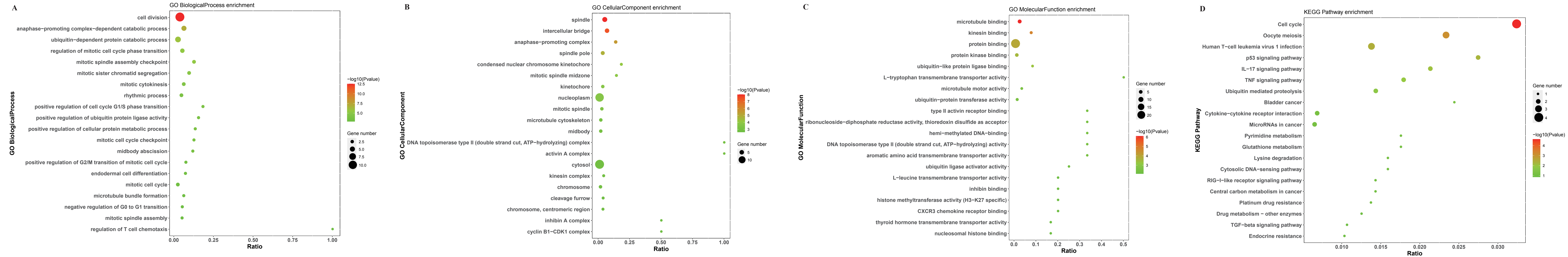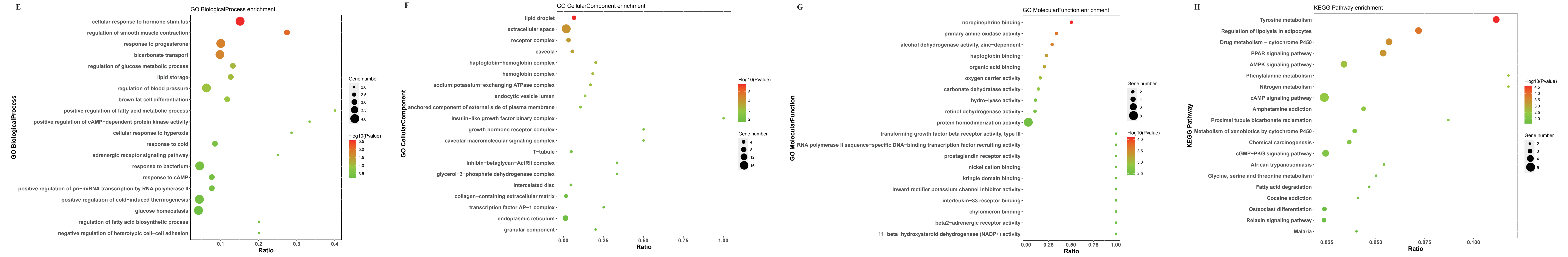

Supplement: Supplementary file 5 [file DataSheet5.PDF]
